# A new mechanistic approach for the further development of a population with established size bimodality

**DOI:** 10.1371/journal.pone.0179339

**Published:** 2017-06-26

**Authors:** Lisa Heermann, Donald L. DeAngelis, Jost Borcherding

**Affiliations:** 1 Institute for Zoology of the University of Cologne, Department of General Ecology, Cologne, Germany; 2 US Geological Survey, Wetlands and Aquatic Research Center, Gainesville, FL, United States of America; Stockholm University, SWEDEN

## Abstract

Usually, the origin of a within-cohort bimodal size distribution is assumed to be caused by initial size differences or by one discrete period of accelerated growth for one part of the population. The aim of this study was to determine if more continuous pathways exist allowing shifts from the small to the large fraction within a bimodal age-cohort. Therefore, a Eurasian perch population, which had already developed a bimodal size-distribution and had differential resource use of the two size-cohorts, was examined. Results revealed that formation of a bimodal size-distribution can be a continuous process. Perch from the small size-cohort were able to grow into the large size-cohort by feeding on macroinvertebrates not used by their conspecifics. The diet shifts were accompanied by morphological shape changes. Intra-specific competition seemed to trigger the development towards an increasing number of large individuals. A stage-structured matrix model confirmed these assumptions. The fact that bimodality can be a continuous process is important to consider for the understanding of ecological processes and links within ecosystems.

## Introduction

The way a population’s size distribution changes with time depends on biotic and abiotic factors influencing growth and development of individuals. Bimodality is a specific case of size-structure that has often been studied (e.g., [1,2]) and that can be created and modified by changing impacts of these factors over time [2].

Besides size-selective mortality, or a broad initial size-distribution, evolving into a bimodal size distribution [3], differential growth is an important mechanism leading to bimodality [4]. Growth rates of individuals can be variously influenced, for instance, by size-dependent factors such as foraging ability or risk avoidance [4], or by individuality in use of food resources [5–7]. Extrinsic factors like environmental heterogeneity can equally well account for development of bimodality via different growth rates, and may be further adjusted through inter-and intra-specific competition and predation (for a review see [4]). While individuals are growing, these interactions of competitive and predatory processes may change distinctly and are an important component of ontogenetic niche shifts [8].

Ontogenetic diet shifts are common in a wide range of taxa; e.g., invertebrates, reptiles and fish (for a review see [9]). In particular, gape-limited predators, such as most piscivorous fish, undergo discrete changes in diet [10], whereby the size-dependence of these diet shifts leads to inter-individual variation in growth, dramatically influencing the size structure of the population [11], potentially enabling bimodality to emerge.

Many studies on the development of bimodality either identified an initial size difference [12] or a single discrete accelerated growth period of one part of the population [1,13] as the origin of within-cohort size variation. However, in a population with a bimodal size distribution, there are often also some individuals with intermediate size. Can these individuals be assigned to the upper edge of the fraction of small individuals or to the lower edge of the large ones? Such a static view may, however, neglect dynamic processes that allow some individuals within the cohort to shift from the smaller to the larger fraction, beyond initial size differences or one-time growth accelerations. One consequence of a broad size-distribution (like bimodality) within an age-cohort can be mainly attributed to differential resource use and the consequential reduction of intra-cohort competition [13,14,15]. One can hypothesize the existence of medium-sized individuals that consume alternative food resources that are not used by the bulk of small or large individuals (e.g., feeding on macroinvertebrates if the majority of the conspecifics prey zooplankton). To test this hypothesis, a system in which a bimodal size distribution with differential usage of food resources by two size-classes already existed was examined to answer (1) if medium-sized individuals differ in food consumption compared to their small and large conspecifics of the size distribution, (2) if this alternative usage of food resources may allow a shift from the fraction of small-sized fish to the fraction of large-sized fish within a bimodal size-distribution, and (3) if abandonment of alternative food consumption (when entering the larger-sized fraction and starting to use their food resources) would then allow more individuals of the small-sized fraction to use this alternative pathway. Such a scenario would provide evidence that pathways exist for a continuous shift from the small to the large fraction within a bimodal age-cohort. A stage-structured matrix model was analysed numerically for the small size-cohort to support findings of the field study and further understand the mechanism behind a shift from the small to the large fraction within a bimodal age-cohort.

## Material and methods

### Study site

Field experiments were conducted in two ponds; pond 1 (P1) 0.6 ha and one pond 2 (P2) 0.7 ha in size, at a fish farm in Lohmar, Germany (50°49'33.00''N 7°12'59.42''E; for more detailed description of the ponds see [16]). The ponds are connected via overflows, assuring that they did not differ in abiotic parameters, such as temperature, oxygen content and conductivity (t-test, all P >0.05; see [17]). The present study is part of a larger investigation on the development of cannibalism. In the context of this larger study, in spring, the ponds were stocked with adult perch and bream (not further considered in this study) in April 2006 (for further details see [17,18]). Before stocking, the ponds were fishless. Adult perch stocked originated from the same population to minimize genetic differences which might, for example, influence growth rates [19]. After spawning, adult fish were removed from the ponds to guarantee undisturbed development of the offspring. A few weeks after hatching some YOY perch first became early piscivores (preying on bream larvae), thereby benefiting from accelerated growth [18]. Shortly after becoming piscivorous, these perch were large enough to cannibalise their smaller conspecifics, which led to establishment of two size-cohorts, a large-bodied cannibalistic and a small-bodied plankti-/macroinvertivorous cohort [18]. At this time the present study started, which means that these two size-cohorts were already present at the beginning of this investigation. Perch density within the first 60 days after perch hatching (before this experiment) did not differ between ponds (t-test P >0.05). For further explanation on how densities were calculated see S1 File.

### Sampling

Starting in mid-June, zooplankton and macroinvertebrates were sampled weekly and biweekly, respectively. After size measurement and counting of organisms, biomass was determined from published length–mass equations and expressed as mg wet weight L^-1^ for zooplankton samples, g wet weight m^-2^ for sediment macroinvertebrates and catch per unit of effort (CPUE) [g section^-1^] for macroinvertebrates in the vegetation. For further details on sampling of zooplankton and macroinvertebrates refer to S2 File.

Perch were sampled monthly using electro-fishing and gillnetting with multi-mesh-sized gillnets (for further details see [16]). Immediately after capture fish were killed with an overdose of metomidate hydrochlorid (Aquacalm ®) and deep-frozen for further analysis. The experiments complied with the current laws of Germany and were approved by the ethic commission of the University of Cologne. All sampling procedures were reviewed and specifically approved as part of obtaining the field permit. The owner of the experimental ponds gave his permission to conduct our studies there. The field studies did not involve endangered or protected species. In the laboratory fish analysed were randomly chosen from all sizes of caught perch, kept separated and registered individually. The length (total length, TL) (to nearest 0.5 mm) of each perch was recorded and each perch was photographed for morphometric analysis using a digital camera [20]. Subsequently, stomach content was analysed. Perch with empty stomachs (4–19% of all perch analysed) were excluded from further analyses, leaving 38–70 individuals per sampling date. The food spectrum of each perch was expressed as the weight percentage composition of food items identified to genus level [21]. For perch of the last sampling date in October the sex and the stage of maturation were documented. As perch had been registered individually, it was possible to assign each photo and its morphometric information to the corresponding stomach content analysis and where applicable stage of maturation of individual perch.

### Data analysis and statistics

For further analysis of stomach contents, prey items were assigned to three groups; zooplankton, macroinvertebrates and fish, and data were expressed as wet weight percentage composition of these three prey categories in perch stomachs. Differences in diet composition of perch were determined by the Bray–Curtis similarity index [22]; i.e., by hierarchical clustering, using the obtained percentage weight data [23]. Data were clustered through group average linking, after which significant differences between samples were calculated by the non-parametric test of similarity (one-way ANOSIM). To illustrate the clustering, an MDS (non-metric multidimensional scaling) plot was drawn for a similarity level of 30% and a stress of 0.01. All cluster analyses were done with PRIMER v6 (Plymouth Routines in Multivariate Ecological Research, Roborough, Plymouth, UK). The diet spectrum of fish assigned to one cluster consisted of at least 50% of the corresponding resource; e.g., fish which had fed on 51% macroinvertebrates were clustered into the macroinvertivorous cohort.

The mean TL ± standard deviation of fish in each cluster was computed. Difference in TL was tested using Student’s t-tests for June and July and one-way ANOVA and Bonferroni post hoc tests for pairwise comparison for the other sampling dates. Growth was calculated as the difference of mean length in each cluster per day [mm day^-1^]. All statistical tests referring to sizes of perch were performed with SPSS 23 (IBM, Armonk, New York, USA).

For morphometric analysis photos of perch and tpsDigit and tpsUtility software by Rohlf (available at: http://life.bio.sunysb.edu/morph/) were used to digitize fourteen landmarks on the perch’s left side as well as a scale (for details see [20]). Perch were grouped according to their assignment to different clusters (c.f. cluster analysis above), after which the Integrated Morphometrics Package (IMP) developed by Sheets (available at: http://www3.canisius.edu/~sheets/morphsoft.html) was used for further morphometric analysis. The differences of shapes between groups (clusters) were analysed using a canonical variates analysis (CVA) and, based on the Wilk’s lambda value at a p < 0.05 level of significance, the CV axes were tested for significance. The shape change was graphically illustrated by vectors on landmarks. As fish differed in size, potential morphological differences were examined for allometric effects. If allometric effects could be detected, shapes were standardized by regressing each group on size separately, thereby removing the variance attributable to size and leaving the shape variation that was independent of size differences. For a more detailed description of the morphometric analysis, the standardisation process and the software used for each calculation; see [20].

### Modelling approach

To further understand how divergent resource use allows a shift from the small-bodied to the large-bodied fraction within a bimodal size-distribution of YOY fish, and to what extent this pathway is open to the initially small individuals, a stage-structured matrix model for YOY fish was used to compute YOY dynamics. The, which is described in more detail in S3 File, model is intended only to demonstrate the mechanisms by which the shifts can occur, and not to attempt to rigorously predict the empirical data. To keep the model simple, we started with an initially small–bodied cohort. We modelled three resources, zooplankton, macroinvertebrates and prey-fish, and enabled diet shifts of YOY from one resource to another. The cohort is divided into 300 stages (weight classes) to obtain a high resolution as growth rate is sensitive to weight differences in YOY perch. Thus, sufficient resolution to describe the continuous variation of weights that are expected in a cohort, was guaranteed. Each stage was associated with a length and weight of the YOY fish in that stage class. These stages can be considered ‘micro-stages’, as they don’t correspond to distinct physiological stages, but only to incremental differences in length and weight. The probability of fish advancing from stage class to stage class in each time step is less than 1, and depends on the amount of available prey. For parameters used in the model see S3 File. The model was calculated with Matlab (MathWorks, Natick, Massachusetts, USA).

The cohort of YOY perch is divided into three consumer groups: planktivores (*B1*), macroinvertivores (*B2*), and piscivores (*B3*), with:

$$\begin{array}{l} B1=[B1_{1}(t),B1_{2}(t),\;\ldots ,B1_{i}(t),\;\ldots ,\;B1_{300}(t)]\\ B2=[B2_{1}(t),B2_{2}(t),\;\ldots ,B2_{i}(t),\;\ldots ,\;B2_{300}(t)]\\ B3=[B3_{1}(t),B3_{2}(t),\;\ldots ,B3_{i}(t),\;\ldots ,\;B3_{300}(t)] \end{array},$$

where *B1*_*i*_*(t)*, *B2*_*i*_*(t)*, and *B3*_*i*_*(t)* are, respectively, the numbers of individuals of the three consumer groups in each of the 300 stages. The simulation keeps track of the YOY through their growing season. These YOY start as purely planktivores (*B1*) with an initial stage distribution (length and weight). At some point a fraction attains a stage with great enough length to move into the macroinvertivore group (*B2*), and eventually some fraction of those grow to a large enough length to advance to the piscivore group (*B3*).

These stage-structured cohort dynamics for the three consumer groups is entirely governed by a Markov chain matrix model:

BJ(t+1)=A•BJ(t)(J=1,2,or3)

where *A*, which describes unidirectional growth in size, is:

$$A=\left|\begin{array}{cccccccccc} a_{11} & 0 & 0 & . & 0 & 0 & 0 & . & 0 & 0\\ a_{12} & a_{22} & 0 & . & 0 & 0 & 0 & . & 0 & 0\\ 0 & a_{23} & a_{33} & . & 0 & 0 & 0 & . & 0 & 0\\ . & . & . & . & . & . & . & . & . & .\\ 0 & 0 & 0 & . & a_{ii} & 0 & 0 & . & 0 & 0\\ 0 & 0 & 0 & . & a_{i,i+1} & a_{i+1.i+1} & 0 & . & 0 & 0\\ 0 & 0 & 0 & . & 0 & a_{i+1,i+2} & a_{i+2.i+2} & . & 0 & 0\\ . & . & . & . & . & . & . & . & . & .\\ 0 & 0 & 0 & . & 0 & 0 & 0 & . & a_{299,299} & 0\\ 0 & 0 & 0 & . & 0 & 0 & 0 & . & a_{299,200} & a_{300,300} \end{array}\right|$$

where *a*_*i*,*i+1*_ represents the fraction advancing from a given stage class *i* to age class *i*+1, and *a*_*i*,*i*_ represents the amount remaining in stage *i*. The elements *a*_*i*,*i*_ and *a*_*i*,*i+1*_ differ depending on which of the three consumer groups the YOY are in at a given time step. The amount initially in stage *i* that advances to the next stage, *i*+1, during a time step is

$$a_{i,i+1}=surv*advance(prey)$$

where *surv* is a fixed constant survival rate for each time step and *advance(prey)* is the fraction of the survivors that advance to the next stage class, which is a function of prey density. The fraction of those initially in stage class *i* that remain there during the next time step is

$$a_{i,i}=surv*(1-advance(prey))$$

For simplicity, we assume that the type of prey being consumed does not affect survival, *surv*, but that it does affect advancement, through the functions;

$$\begin{array}{l} advance(zooplankton)=\frac{q_{1}Z}{q_{2}+Z}\\ advance(benthic)=\frac{q_{3}M}{q_{4}+M}\\ advance(preyfish)=\frac{q_{5}F}{q_{6}+F} \end{array}$$

where *Z*, *F*, and *M* are the current available biomasses of zooplankton, prey fish, and macroinvertebrates, respectively. The fraction of advance to the next stage depends on the density of available prey. The *q*_*i*_s are constants that can be chosen to corresponding to given assumptions on how the prey biomasses of each type affect advances to the next stage.

In this case it was assumed that YOY perch feeding on fish reach the highest growth rates, followed by those feeding on macroinvertebrates, while planktivorous perch have the lowest growth rates (i.e., *q*_*3*_ > *q*_*5*_ > *q*_*1*_). Additionally, the growth rates also depend on the concentrations prey, which are changing through time. The *q*_*i*_’s are calibrated to give realistic rates of growth. Prey densities are described by the dynamic equations:

$$\begin{array}{l} \frac{dZ}{dt}=r_{z}\left(1-\frac{Z}{K_{z}}\right)-\frac{f_{z}Z\sum_{i=1,300}Weight_{i}B1_{i}}{1+f_{z}hZ}\\ \frac{dM}{dt}=r_{m}\left(1-\frac{M}{K_{m}}\right)-\frac{f_{m}M\sum_{i=1,300}Weight_{i}B2_{i}}{1+f_{m}hM}\\ \frac{dF}{dt}=r_{f}\left(1-\frac{F}{K_{f}}\right)-\frac{f_{f}F\sum_{i=1,300}Weight_{i}B3_{i}}{1+f_{f}hF} \end{array}$$

where *Weight*_*i*_ is the weight of perch in stage class *i*, *f*_*z*_, *f*_*m*_, and *f*_*f*_ are feeding rates, and 1/*h* is the maximum digestion of biomass per unit time by the perch. *r*_*z*_, *r*_*m*_, and *r*_*f*_ are renewal rates of zooplankton, macroinvertebrates and prey-fish, and *K*_*z*_, *K*_*m*_, and *K*_*f*_ are the carrying capacities. Finally, perch are assumed to be able to switch prey types when they reach certain threshold stage classes, and switch then with certain probabilities each time step. At this time there are only two switches, from planktivory to macroinvertivory and from macroinvertivory to piscivory.

*Stage_transfer_to_inverts* = length at which planktivores can start to switch to macroinvertivory
*Stage_transfer_to_fish* = length at which macroinvertivores can start to switch to piscivory
*Fraction_transfer_to_inverts* = fraction of planktivores switching to macroinvertivory each time step
*Fraction_transfer_to_fish* = fraction of macroinvertivores switching to piscivory each time step

## Results

Zooplankton biomass was low, ranging between 0.1 and 1.5 mg L^-1^, while macroinvertebrates sampled in the sediment ranged between 5.4 and 10.5 g m^-2^. For macroinvertebrates in the vegetation a CPUE (g section^-1^) of 0.02 to 0.05 was found (S2 File).

The density of perch before this experiment declined from a mean for both ponds of 14 Ind m^-2^ (± 3 SD) right after hatching (09 May) to a mean of 5 Ind m^-2^ (± 0 SD) about 60 days after perch hatch (22 June, start of this study, S1 File). The calculated density of perch in the ponds at the end of this study when the ponds were completely emptied was similar (mean 5 ± 3 SD), suggesting that only minor changes of perch density occurred during this study (S1 File).

**Fig 1:**
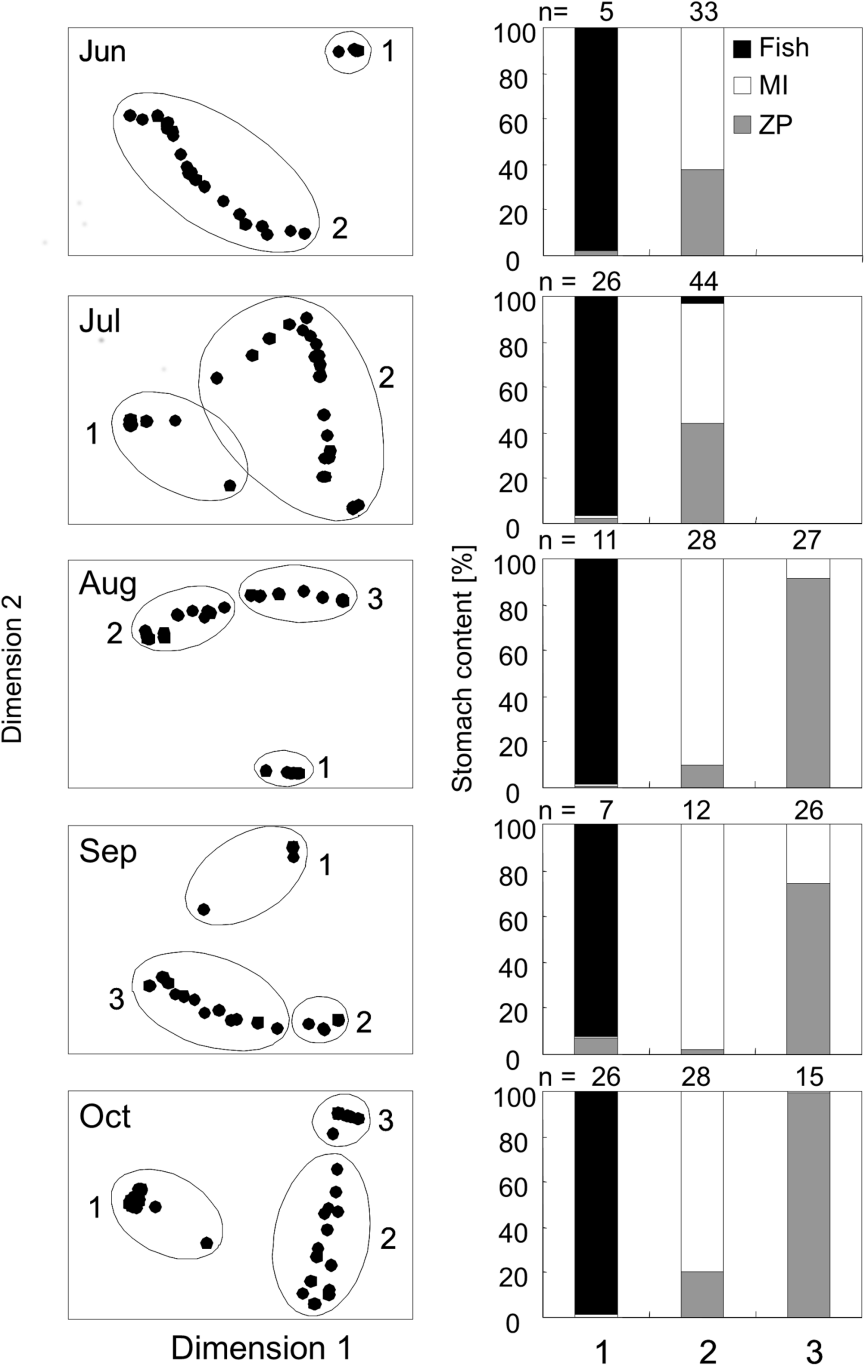
Cluster analysis based on diet of Eurasian perch as well as stomach content of perch. Left panel: non-metric multidimensional scaling (MDS) plot of hierarchical cluster analysis based on Bray–Curtis similarity index of stomach content analysis of Eurasian perch (*Perca fluviatilis*). The MDS plot was drawn for a similarity level of 30% and a stress of 0.01. Right panel: Stomach content [% of wet biomass] of perch in clusters 1 to 3 throughout the season. MI = Macroinvertebrates, ZP = Zooplankton.

Stomach content analysis showed that perch consumed fish, macroinvertebrates or zooplankton (Fig 1). More precisely, perch that consumed fish were found to be cannibals preying on their smaller conspecifics. Macroinvertebrate prey were mainly ephemeropterans, zygopterans and chironomids, while the zooplankton that was consumed predominantly consisted of daphnids, copepods and small daphnoids such as *Bosmina*.

Cluster analysis based on stomach content analysis split up the perch population during June and July into two clusters, where perch in cluster 1 fed on fish, while perch in cluster 2 consumed a mixed diet of macroinvertebrates and planktonic organisms (Fig 1). In August, September and October cluster analysis indicated three clusters. Perch in cluster 1 consumed fish, perch in cluster 2 principally preyed on macroinvertebrates, and perch in cluster 3 fed mainly on zooplankton (Fig 1). An overview of the variation of main food resources used by perch in the different clusters can be found in S1 Table.

**Fig 2:**
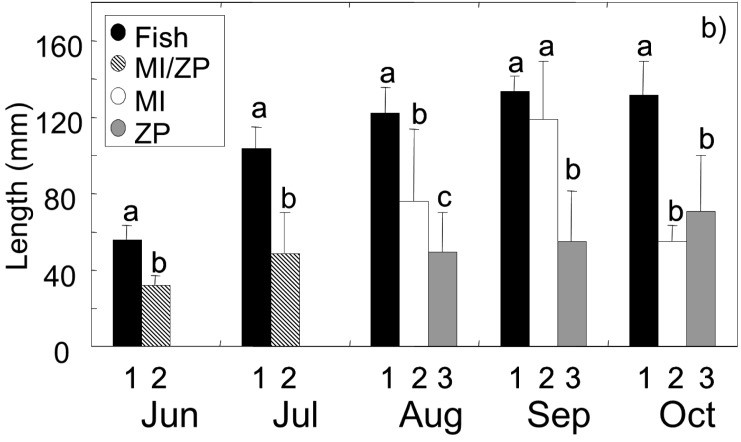
Mean length [mm] of perch in different clusters. The calculation of clusters was based on stomach content analysis (Fig 1). Different shades indicate the food resource mainly used by perch in the cluster, letters indicate significant differences of length between clusters (based on Student’s t-tests for June and July and one-way ANOVA and Bonferroni post hoc tests for pairwise comparison for the other sampling dates). Error bars give the standard deviation (SD). MI = macroinvertebrates, ZP = zooplankton.

In June and July perch in cluster 1 (fish-consuming) were significantly larger (Student’s t-tests: Jun: t_5,33_ = 9.02, p<0.001; Jul: t_26,44_ = 12.2, p<0.001) than perch of cluster 2 (consuming macroinvertebrates and plankton) (Fig 2). In August fish-consuming perch in cluster 1 were the largest, while macroinvertebrate-consuming perch in cluster 2 had an intermediate size and plankton-consuming perch of cluster 3 were the smallest. Here all three clusters significantly differed from each other in size (ANOVA: F_2,63_ = 26.1, p<0.001; Bonferroni post hoc test: 1>2>3, p at least <0.01). In September piscivorous perch (cluster 1) and macroinvertivorous perch (cluster 2) no longer differed in size (ANOVA: F_2,42_ = 39.0, p<0.001; Bonferroni post hoc test: 1 = 2, p>0.05), but both together formed the larger perch size group that was significantly larger than planktivorous perch in cluster 3 (ANOVA: F_2,42_ = 39.0, p<0.001; Bonferroni post hoc test: 1 and 2>3, p<0.001). In October, piscivorous perch (cluster 1) were significantly larger than perch in cluster 2 and 3 (ANOVA: F_2,66_ = 72.1, p<0.001; Bonferroni post hoc test: 1>2 and 3, p<0.001), while the size of macroinvertivorous (cluster 2) and planktivorous (cluster 3) perch was the same (ANOVA: F_2,66_ = 72.1, p<0.001; Bonferroni post hoc test: 2 = 3, p>0.05) (Fig 2).

The analyses of sex and maturity of perch caught in October revealed that in total (independent of size) 53.6% of the 69 perch analysed were females. In the large piscivorous size-cohort 57.7% of all perch were males. Males and females in the large size-cohort did not differ in size (TL males: 138.1 ± 14.2 SD, TL females: 126.6 ± 17.1 SD; Student’s t-tests: t_11,15_ = 1.77, p>0.05). 93.3% of male perch in the large-size-cohort were mature, while this was the case for only one female (9.1%).

During the period from June to July, piscivorous perch (cluster 1) grew, on average, 1.7 mm day^-1^, after which their growth rates continuously decreased until October, when growth could no longer be detected (Table 1). From June to July, small perch (cluster 2), that fed on a mixed diet of macroinvertebrates and zooplankton grew 0.6 mm day^-1^. After this period, perch in cluster 2 (now solely feeding on macroinvertebrates) increased mean growth rates to a maximum of 1.4 mm day^-1^ from August to September. From September to October, mean growth rates in cluster 2 seemed to dramatically decrease to -2 mm day^-1^. Here, however, negative growth rates illustrate that perch in cluster 2 have been much larger in September than in October (Table 1) indicating that largest perch had performed a diet shift, while only the smaller perch remained in cluster 2, still consuming macroinvertebrates. Small planktivorous perch (cluster 3 from August and onwards) grew only minimally with a mean rate of 0.3 mm day^-1^ (maximal growth rate = 0.5 mm day^-1^; Table 1).

**Table 1:** Growth rates [mm day^-1^] of perch in different clusters.

	Cluster1	Cluster2	Cluster3
**Jun-Jul**	1.71 (F)	0.60 (ZP / MI)	
**Jul-Aug**	0.75 (F)	1.08 (ZP / MI)	
**Aug-Sep**	0.38 (F)	1.42 (MI)	0.18 (ZP)
**Sep-Oct**	-0.08 (F)	-2.04 (MI)	0.52 (ZP)

Next to the growth rates the food resource used by perch is indicated in brackets. F = fish (more precisely cannibals), ZP = zooplankton, MI = macroinvertebrates. The calculation of clusters was based on stomach content analysis (Fig 1).

**Fig 3:**
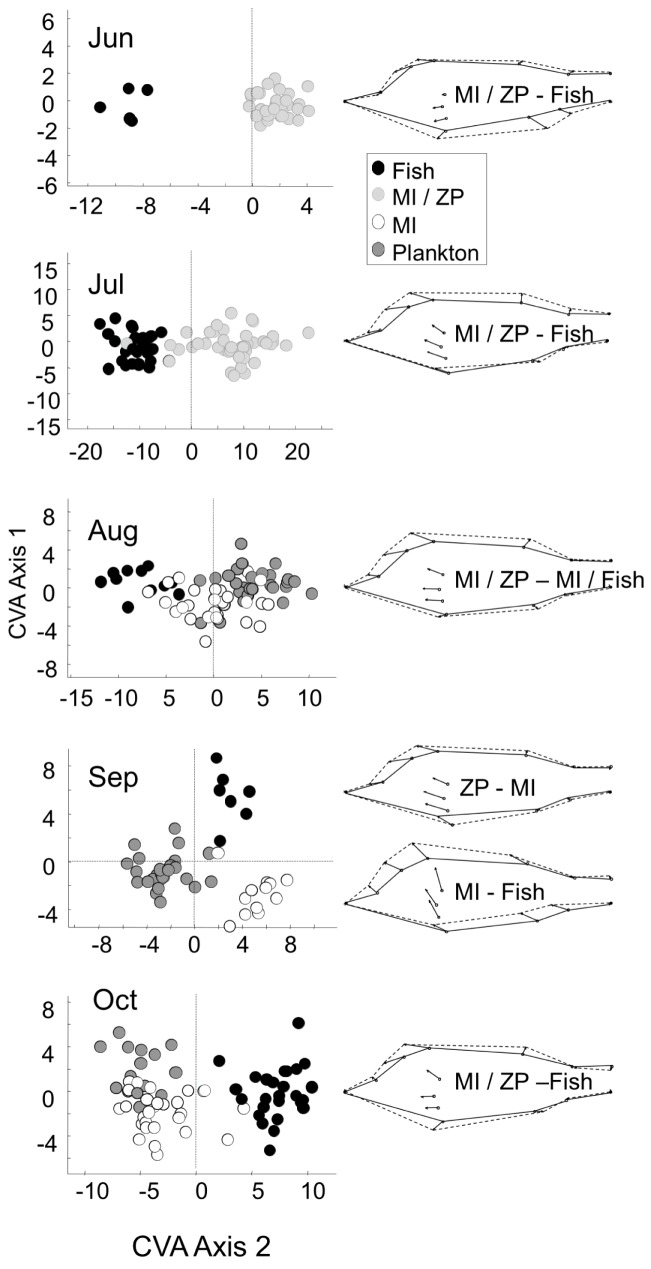
Morphometric analysis of perch. Left panel: canonical variates scores of perch in different clusters (the calculation of clusters was based on stomach content analysis, Fig 1) throughout the season, depicted along the first and the second canonical variates axis. Different shades indicate the food resource mainly used by perch in the cluster. Significant axes separating the morphs are shown as lines (one axis except for September when two significant axes were found). Right panel: Shape change correlated with the first CVA axis between perch in different clusters, obtained by regressing the shape on the CVA axis scores, depicted as growth vectors. The shape change depicted always starts from the lower trophic level (e.g., the change from planktivorous to piscivorous perch). Lettering inside the shapes of perch indicates which groups have been compared. MI = macroinvertebrates, ZP = zooplankton.

Morphometric analysis resulted in two morphs in June and July (one significant axis: Jun: λ = 0.09, χ^2^ = 58.5, d.f. = 24, eigenvalue = 10.4, p<0.001; Jul: λ = 0.26, χ^2^ = 76.4, d.f. = 24, eigenvalue = 2.91, p<0.001), one which fed on fish, the other which fed on macroinvertebrates and plankton (Fig 3). Also in August one significant axis was found (λ = 0.25, χ^2^ = 72.1, d.f. = 48, eigenvalue = 1.86, p<0.05) separating piscivorous from planktivorous perch, while the morph of macroinvertivorous perch was in-between the planktivorous and piscivorous morph, but not significantly different from any of those. However, in September all three morphs, the piscivorous, macroinvertivorous and planktivorous, were separated along two significant axes (axis 1: λ = 0.05, χ^2^ = 94.8, d.f. = 48, eigenvalue = 4.6, p<0.001, explaining 61% of the variance; axis 2: λ = 0.25, χ^2^ = 42.0, d.f. = 23, eigenvalue = 2.96, p<0.01, explaining 39% of the variance). In October again only two morphs could be detected (one axis: λ = 0.1, χ^2^ = 125.8, d.f. = 48, eigenvalue = 45.6, p<0.001), separating the piscivorous perch from the macroinvertivorous and planktivorous perch (Fig 3). The difference in shape between morphs was similar for all sampling dates. When compared in direction from the lower trophic position towards the higher one (e.g., from planktivorous to piscivorous), perch became deeper-bodied and developed a shorter head (pelvic fins and operculum were shifted anteriorily; Fig 3).

**Fig 4:**
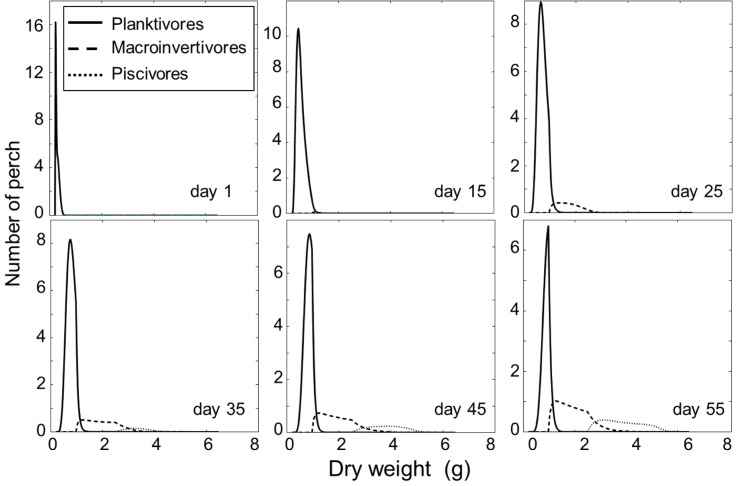
Weight distribution (g dry weight) of young-of-the-year perch simulated in the stage-structured model. Model output is shown for day 1, 15,25,35,45 and 55 of the growth season. Only the small size-cohort was modelled, where perch can be planktivorous, macroinvertivorous or piscivorous.

Initial conditions (day 1) of the stage-structured model illustrate the small-bodied size-cohort, which is planktivorous (day 1, Fig 4). Model results of day 15 illustrate the situation that was documented for the small size-cohort at the start of this study in the field. There were no big size-differences within the cohort and perch mainly fed on zooplankton but to a smaller extent also on macroinvertebrates. Already ten days later (day 25) macroinvertivorous perch accelerate in growth and can then switch to piscivory on day 35. The amount of zooplanktivorous individuals shifting to a macroinvertivorous diet continuously increases with more and more individuals becoming piscivorous (day 35–55, Fig 4).

## Discussion

Using the example of Eurasian perch, this study showed that, in a population with an already existing bimodality, the size distribution of the population changes further dynamically. Induced by distinct shifts in diet, these changes are clearly accompanied by morphological alterations.

There are two possible alternative mechanisms behind this development.

1) To grow from the small planktivorous/macroinvertivorous cohort into the large cannibalistic size-cohort, a group of small perch first shifted from a mixed diet of zooplankton and macroinvertebrates to pure macroinvertivory in August (first escapees) (see Fig 2 and S1 Fig). This was about 4 months after hatching and also clearly after the period when bimodality in the system had already been established due to predation on bream larvae by some of the YOY perch, mainly in May [24]. When the aforementioned group of small perch had shifted to a macroinvertivorous diet, the remaining perch of the small size-cohort fed mainly on zooplankton (instead of feeding on a planktivorous/macroinvertivorous diet). The diet shift of this first group of escapees was clearly reflected in accelerated growth and morphological shape changes. Accelerated growth of this medium-sized fraction of the age-cohort was great enough that they reached their larger conspecifics in size within 2 months and finally performed their second diet shift, becoming piscivorous/cannibalistic. After the escapees from the small size-cohort had left the macroinvertivorous niche and become cannibals, most of the remaining small perch performed a distinct diet shift towards macroinvertivory.

2) Alternatively, the development of the macroinvertivorous group in August (Fig 2) might not have originated from a part of the small cohort shifting to macroinvertivory and benefitting from accelerated growth. Instead, a part of large cannibalistic perch might have interrupted piscivory and shifted to macroinvertebrates, thus increasing mean length of this group. This process could have continued in September with more piscivorous perch switching to macroinvertivory, further augmenting the group’s mean length. For both months the diet shift of large perch could have been accompanied by morphological shape changes. Following this scenario, the interpretation of results in October would show that many, if not all, large perch now performed a diet shift back to cannibalism and only the small perch which were already macroinvertivorous in August remain in this group. Growth of these macroinvertivorous small perch would then be absent (small perch in July already have the same size as small perch in October). This second shift of large perch back to cannibalism would be reflected in a second change in morphology towards the piscivorous morph.

Supporting the possibility of the second alternative is the fact that perch is a flexible ontogenetic diet shifter ([25] and references therein) and there are examples (however, very few) of interrupted piscivory of fish [26]. Although the second possible mechanism might occur, we think that it will play only a minor role, for the following reasons. (1) During the whole study small perch were always present in high abundance. When the ponds were emptied, it was found that 95% of all perch were of small enough size to be possible victims of cannibalism. (2) To our knowledge it has never been shown that perch first develop a deep-bodied morph with piscivory, but become lower bodied (not more slender!) again when feeding on macroinvertebrates, and finally develop a deep body while shifting to piscivory another time. This is also not likely, as the development of a deeper body is regarded as the species-specific ontogenetic growth trajectory in juvenile perch [20,27]. We therefore will focus on the first mechanism, which was also confirmed by the stage-structured model, and discuss this one in detail below.

### Diet shifts and morphometry

The performed shifts match widely known descriptions of perch’s ontogenetic diet shifts, where perch change from a planktivorous via a macroinvertivorous to a piscivorous diet [8]. However, diet shifts are usually performed over several years (perch regularly become piscivorous when 2 or 3 years old, [8,10]), while in this case the first group of perch escaping from the small cohort performed both diet shifts within one growing season. Therefore, at times the diet of medium-sized perch significantly differed from that of their smaller and larger conspecifics.

For the first group of escapees the change in consumed food resources was reflected in significant shape changes of juvenile perch. Such changes in morphology are not only documented in the ontogeny of perch [28], but also in many other organisms. They are regularly attributed to changes in the preferred habitat [29], resource use [30,31] or predation pressure [32]. Morphological change could in this study first be detected in as short a time as 4 weeks [33]. Generally, the shift to a higher trophic position (first to macroinvertivory, then to piscivory) was reflected in the development of a deeper body and a shorter head (pelvic fins and operculum moving anteriorily), which was suggested to be the species-specific ontogenetic growth trajectory in juvenile perch, given a sufficient energy intake [20].

### Competition and growth

The scenario of usage of the macroinvertivorous niche by the different size-classes and morphological forms (first and second group of escapees) provides some evidence that intra-specific competition may be an important factor in the whole process of the continuous formation of bimodality. The population density in the experimental ponds used for this study was high (5 Ind m^-2^, S1 File, also see [18]) compared to other systems (e.g., about 0.6 Ind m^-2^, [34]; about 0.3 Ind m^-2^, [11]), and the small size-cohort represented the major part (more than 95%) of the YOY perch population. Additionally, zooplankton was soon depleted after perch hatching [18] and, hence, scarce during the period of this study (S2 File). High population density and low zooplankton resources without any doubt induced strong intra-specific competition within the small size-cohort [6], which is clearly reflected in low growth rates (on average 0.6 mm day^-1^). At high levels of competition and low resource availability, fish were shown to shift to alternative food resources not used by conspecific competitors [6,35,36]. The food resource chosen differed among individuals, suggesting that some individuals are being favoured in terms of growth due to the fact that they feed on a more energetic resource (e.g. macroinvertebrates or fish). Consequently, such a process of resource use diversity with higher growth rates, which may accelerate within short periods [7], would be rather independent of ontogenetic diet shifts. Exactly such a scenario became obvious in this study, as size-differences within the small size-cohort were rather minor, but still only a part of all individuals escaped from the small cohort by shifting to higher trophic levels. On the other hand, and in contrast to zooplankton, macroinvertebrates were not depleted but appeared in amounts similar to (or even higher than) other ponds inhabited by fish (e.g. all over biomass 0.5–8 g m^-2^ at 50 kg ha^-1^ fish, [37]; this study 5–11 g qm^-2^ [note that in this study only sediment organisms!] [18]).

After the macroinvertivorous niche was occupied by escapees from the small size-cohort, remaining small conspecifics solely consumed zooplankton (reflected in minimal growth rates of 0.5 mm day^-1^), giving some evidence that perch that did not perform the diet shift were the weaker competitors for macroinvertebrates and hence were forced to consume the less profitable and still depleted resource zooplankton. In fact laboratory experiments using three-spined stickleback (*Gasterosteus aculeatus* L.) fed with two sizes of *Daphnia* demonstrated that stronger competitors can force subordinates to opt for less profitable food items [38]. On the other hand, the small number of perch that had shifted to macroinvertivory (escapees) now benefited from accelerated growth (maximal 1.4 mm day^-1^), which might be associated with a lower level of experienced intra-specific competition for macroinvertebrates (as not all perch had shifted to macroinvertivory). This is corroborated by a study on largemouth bass (*Micropterus salmoides* Lacépède), indicating a growth advantage of early diet shifters (towards a piscivorous diet) compared to bass becoming piscivorous at later stages [12]. Later, in October, medium-sized perch had left the macroinvertivorous niche and had become piscivorous, thus completing a shift from the small to the large fraction within a bimodal size-distribution. Thereafter, most perch of the small-sized individuals shifted from a planktivorous to macroinvertivorous diet; thus, on their part, reducing experienced intra-specific competition. Consequently, this diet shift of remaining small perch reflects the fact that once competition is no longer influencing the small size-cohort, the pathway for growing into the large size-cohort was potentially cleared.

The stage-structured model supported the mechanism described above. At the beginning of the calculation small perch had only minor size-differences. However at day 15 modelling results showed that some of the small perch start incorporating macroinvertebrates into their diet. These individuals soon benefited from accelerated growth and were finally able to shift to piscivory. The pathway of shifting to macroinvertivory and later to becoming piscivorous, remained valid for other individuals of the small size-cohort. Hence, the model shows that once perch start shifting from macroinvertivory to piscivory, more and more small individuals follow and start feeding on macroinvertebrates; hence, on their part, accelerating growth and catching up in size with the large piscivorous cohort.

As known for many fish species, perch display sexual dimorphism in size, growth and maturation [39–41]. Female perch, for instance, were shown to invest in active feeding [42] to ensure somatic growth and later gonad development. Male perch need less energy to produce sperm and therefore are not as dependent on high energy intake and growth as females [43]. The pathway, as described above allowing fish via accelerated growth to switch from a small size-cohort to a large one could therefore be favoured by female perch. Our results showed that the ratio of males and females caught in October (females: 53.6%) corresponds well to the natural sex ratio of other perch lakes ([42] and references therein). The large piscivorous size-cohort, however, was slightly dominated by males (57.7%). There was no sex-specific difference of perch TL in the large size-cohort; however, almost all male perch were found to be mature (93.3%), while this was only the case for one female (9.1%). These results suggest that the switch of perch from the small to the large size-cohort accompanied by diet shifts and morphological changes is performed by both sexes, although males may grow into the large cohort slightly more often. While both sexes can reach the same size, males directly benefit from the switch into the large piscivorous cohort, as they can mature and thus reproduce after only one growth season, which was also shown in other studies [24].

### Development of bimodality

The change of size variation over time based on differing in individual growth rates within a population has been shown for several animals across different taxa [2]. For instance, in tadpoles (*Rana sylvatica* LeConte), individual differences in foraging ability led to increased size variation within populations under high competition [15]. The origin of bimodality has been attributed to either initial size differences or to one discrete period of accelerated growth in one part of the population [1,12,13,44]. Initial size-differences involve various factors, such as maternal effects [45] or the timing of spawning ([3] and references therein). A competitive environment may enhance the importance of size-independent factors, leading to increased size variation over time being more likely under high competition [13,15].

Most studies on bimodality have shown that once two size-cohorts developed, bimodality further amplifies with time. This amplification was studied for bimodal size-distributions related to factors such as spatial variation in resource availability or size-dependence and temporal variation in availability of resources [2]. If resource availability increases with size, and temporal variations in resource levels exist, an initial size distribution easily develops into bimodality [46]. Size-dependent resource as the origin of bimodal size-distributions has been verified by theoretical approaches and also an amplification of bimodality through time has been demonstrated [1]. The results of former studies indicating the amplification of bimodality revealed that existing size-cohorts, once they have been formed, are rather discrete and individuals of a population do not grow from one cohort into the other. This is also corroborated by a long-term study on Arctic charr (*Salvelinus alpinus* L.) in a Norwegian lake demonstrating that the size-cohorts within a bimodal size-distribution, which had developed after a mass removal of stunted charr, could be tracked down for several years [47].

Generally, the mechanism described in our study, where differences in individual growth rates over time in a competitive environment led to the formation of distinct size-cohorts, resemble former studies examining the development of bimodality [18,24]. In addition, our study showed that the formation of a bimodal size-distribution is a continuous process persisting for at least a whole growth season. The fact that bimodality is not necessarily attributable to one defined incidence (such as different spawning periods leading to an initial size difference or one discrete accelerated growth period of one part of the population) reveals that alternative and more continuous pathways exist for the formation of bimodality. Thereby, the ratio of differently sized individuals within the population continually changes in relatively short time periods. Cohorts differ not only in size but also in morphological, physiological or behavioural traits [16,20,24,48], which transfers into differing positions within and impact on the whole system. Therefore, it is highly important to consider continuously changing size-distributions for a better understanding of ecological processes and links within an ecosystem.

## Supporting information

S1 File Density of perch 60 days after hatching (02 May–22 June) and at the end of this study: Sampling, analyses and results. (PDF)

S2 File Zooplankton and macroinvertebrates: Sampling and results. (PDF)

S3 File The stage-structured model on young-of-the-year perch: Description and model parameters. (PDF)

S1 Table Percentage composition of food resources consumed by perch. Given is the weight percentage composition (± standard deviation) of main food resources for perch assigned to different clusters. The calculation of clusters was based on stomach content analysis (Fig 1). Grey shades illustrate to which clusters perch were assigned to for the different dates. For example in June and July two clusters were found, one with perch feeding on prey-fish, the other one with perch consuming a mixed diet of zooplankton and macroinvertebrates. From August onwards perch were assigned to three clusters (consuming fish, macroinvertebrates or zooplankton). MI = macroinvertebrates, ZP = zooplankton, NA = not existent.
(PDF)

S1 Fig Graphical illustration of diet shifts and morphological development of perch from June to October 2006. Letters in the fish or directly beside them indicate the food resource consumed. F = fish-eating (piscivorous), MI = macroinvertivorous, ZP = zooplanktivorous. The colours of fish which are explained in the legend box stand for the morphological group fish belong to. At times there might be no overlap of resources consumed and the morphological group. This is because it takes some time for the morph to change after diet shifts. Asterisks indicate an intermediate, but not significantly different morph. E.g., a morph whose traits are in between the one of piscivores and zooplanktivores.
(DOCX)
