# Supplementary material for: A new mechanistic approach for the further development of a population with established size bimodality
Source: PLoS One. 2017 Jun 26;12(6):e0179339. doi: 10.1371/journal.pone.0179339 (PMC5484486; doi:10.1371/journal.pone.0179339)
Supplement: S1 File — (PDF) [file pone.0179339.s001.pdf]

S1 File: Density of perch 60 days after hatching (02 May – 22 June) and at the end of this study: sampling, analyses and results

At the end of the experiment, all fish were extracted from the ponds, all ponds being emptied completely to measure the total wet weight biomass of fish per pond. All fish were weighed before three to four subsamples (about 200 to 700 fish per sample) were taken to measure the fish (to the nearest 0.5 mm), in order to obtain a length-frequency distribution (LFD). Length-weight regressions of these data and the total wet weight biomass of fish per pond were used to calculate the number of individuals per pond as Ind m^{-2}. Perch density within the first 60 days of perch life was calculated based on electro-fishing point abundance data. Therefore fish size, current density, water temperature and conductivity were taken into account in non-linear regressions and the density calculated as Ind m^{-2} (for further detail see Scharbert and Borcherding 2013; Heermann et al. 2014). Due to gear-selectivity of the different methods used to catch fish in this study (electrofishing and gillnetting) it was not possible to calculate overall perch density for remaining time periods.

S1 Table: Density of perch [Ind m^{-2}] 60 days after hatching (02 May – 22 June) and at the end of this study (emptying of ponds on 14 October) in pond 1 (P1) and pond 2 (P2). Periods of this study are highlighted in grey. Densities of 02 May to 06 June appeared before this study but are shown to illustrate the development of the perch population over the whole growing season.

	**P1**	**P2**
**02 May**	10	10
**09 May**	17	11
**15 May**	11	12
**25 May**	5	8
**30 May**	5	5
**06 Jun**	10	10
**22 Jun**	5	5
**14 Oct**	3	8

References:

Scharbert A, Borcherding J. Relationships of hydrology and life-history strategies on the spatio-temporal habitat utilisation of fish in European temperate river floodplains. Ecol Indic. 2013;29: 348-360.

Heermann L, Scharf W, van der Velde G, Borcherding J. Does the use of alternative food resources induce cannibalism in a size-structured fish population? Ecol Freshwat Fish. 2014;23: 129-140.
