# Supplementary material for: A new mechanistic approach for the further development of a population with established size bimodality
Source: PLoS One. 2017 Jun 26;12(6):e0179339. doi: 10.1371/journal.pone.0179339 (PMC5484486; doi:10.1371/journal.pone.0179339)
Supplement: S2 File — (PDF) [file pone.0179339.s002.pdf]

S2 File: Zooplankton and macroinvertebrates: sampling and results.

Starting in mid-June, zooplankton and macroinvertebrates were sampled weekly and biweekly, respectively. Zooplankton and macroinvertebrates were taken parallel to fish sampling, however not exactly at the same locations in the pond to ensure undisturbed capture of a representative fish sample. On each sampling date, within one hour during the daytime, six samples of zooplankton were taken with a Schindler-Patalas trap (5L, mesh size 100 µm) in each pond pond within the first metre of the water column. Macroinvertebrates were sampled using a Birge-Eckmann grab at three representative locations in each pond and sieved through a net of 1 mm mesh size. A dip net (1 mm mesh size) was used to catch macroinvertebrates living in the vegetation. Therefore at three spots the net was moved back and forth about 30 cm above the bottom along a 1-m stretch. Zooplankton and macroinvertebrate organisms were counted and identified to genus level. Ten individuals of each taxon were measured, the biomass was determined from published length–mass equations and expressed as mg wet weight L^{-1} for zooplankton samples, g wet weight m^{-2} for sediment macroinvertebrates and as catch per unit of effort (CPUE) [g $section^{-1}$] for macroinvertebrates in the vegetation. For further details on sampling of zooplankton and macroinvertebrates refer to Heermann et al. 2014.

After checking for homogeneity of variances (F-test, $p>0.05$), data of zooplankton (ZP) and macroinvertebrates (MI) sampled in sediment and vegetation were tested for differences between the two sampling ponds over the season using Student’s t-tests. As significant differences could not be detected (ZP: $t = -1.54$, $df = 9.16$, $P < 0.05$; MI sediment: $t = -0.32$, $df = 5.62$, $P < 0.05$, MI vegetation: $t = -0.45$, $df = 5.45$, $P < 0.05$) mean values were calculated for each date and resource. All statistical tests referring to food resources were performed with software R (www.r-project.org).

S2 Table: Mean biomass (± standard deviation) of zooplankton (ZP) [mg wet weightL^{-1}], macroinvertebrates (MI) in the sediment [g wet weightm^{-2}] and catch per unit effort (CPUE) of MI in the vegetation [g$section^{-1}$] of both ponds (P1 and P2) from June to September 2006.

	ZP [mg wet weight L^{-1}]		MI bottom [g wet weight m^{-2}]		MI vegetation [g $section^{-1}$]	
**22 Jun**	0.08	±0.07	5.40	±2.92	0.03	±0.04
**3 Jul**	0.17	±0.16				
**18 Jul**	1.53	±2.03	10.48	±1.62	0.03	±0.02
**30 Jul**	0.67	±0.34				
**14 Aug**	0.84	±0.60	7.16	±3.23	0.02	±0.01
**28 Aug**	0.60	±0.01				
**13 Sep**	1.43	±0.08	5.43	±3.43	0.05	±0.01

References:

Heermann L, Scharf W, van der Velde G, Borcherding J. Does the use of alternative food resources induce cannibalism in a size-structured fish population? Ecol Freshwat Fish. 2014;23: 129-140.
