# Supplementary material for: A new mechanistic approach for the further development of a population with established size bimodality
Source: PLoS One. 2017 Jun 26;12(6):e0179339. doi: 10.1371/journal.pone.0179339 (PMC5484486; doi:10.1371/journal.pone.0179339)
Supplement: S3 File — (PDF) [file pone.0179339.s003.pdf]

S3 File: The stage-structured model on young-of-the-year perch: description and model parameters.

Here we elaborate further on the model description of the main text.

Purpose

The purpose of the model is to help understand the further dynamics of a cohort of young-of-the-year (YOY) perch that has established a bimodal size distribution, with the smaller subcohort of perch continuing to feed on zooplankton and the larger subcohort feeding on smaller fish prey. In particular, the model is intended to show that the larger individuals within the small-body-sized cohort, by switching to a third prey type, benthic macroinvertebrates as they reach a threshold size. Their subsequent rapid growth rate allows them to almost catch up with the piscivorous perch. Therefore, the benthic macroinvertebrates act as a 'bridge', allowing a continuous transition of planktivorous perch to piscivorous perch after the initial establishment of bimodality.

Entities and their properties

The cohort of YOY perch is divided into three functional groups according to consumptions: planktivores (*B1*), macroinvertivores (*B2*), and piscivores (*B3*), with each consisting of 300 possible stages, representing size classes;

$$\mathbf{B1} = [B1_1(t), B1_2(t), \ldots, B1_i(t), \ldots, B1_{300}(t)]$$
$$\mathbf{B2} = [B2_1(t), B2_2(t), \ldots, B2_i(t), \ldots, B2_{300}(t)]$$
$$\mathbf{B3} = [B3_1(t), B3_2(t), \ldots, B3_i(t), \ldots, B3_{300}(t)],$$

where $B1_i(t)$, $B2_i(t)$, and $B3_i(t)$ are, respectively, the numbers of individuals of the three consumer groups in each of the 300 stages. The simulation keeps track of the YOY through their growing season. The cohort is divided into 300 stages (weight classes) to obtain a high resolution as growth rate is sensitive to weight differences in YOY perch. Thus, sufficient resolution to describe the continuous variation of weights that are expected in a cohort, was guaranteed. Each stage was associated with a length and weight of the YOY fish in that stage class. These stages can be considered 'micro-stages', as they don't correspond to distinct physiological stages, but only to incremental differences in length and weight. The weight of individuals in each stage *j* is described by the equation

$Weight_j = 0.2 + 0.05 * \left(6.*(1 - e^{-0.015*j})\right)^3$ grams dry weight (or g dW).

(see Fig S4.1).

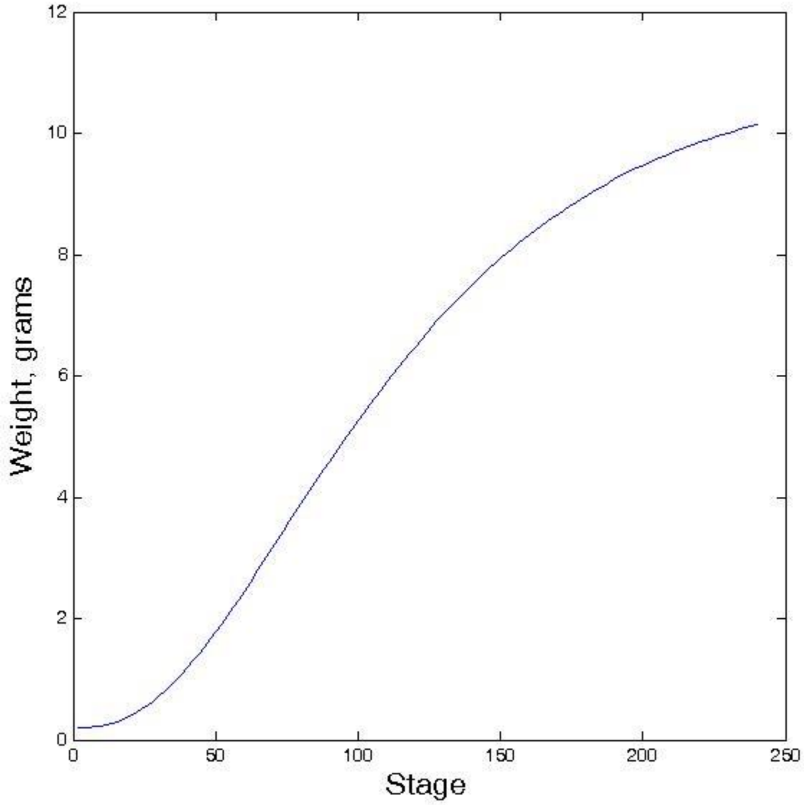

Figure S4.1. Weight in grams per stage for perch population

The other entities in the model are the amounts of prey of each type; zooplankton, benthic invertebrates, and small prey fishes.

Scales

The simulation is assumed to take place in a 10 square meter area and to have daily time steps over the period June 15 to October 15.

Processes

These stage-structured cohort dynamics for the three consumer groups is entirely governed by a Markov chain matrix model:

$$BJ(t+1) = A \bullet BJ(t)$$

(J = 1, 2, or 3)

where A, which describes unidirectional growth in size, is:

$$A = \begin{vmatrix} a_{11} & 0 & 0 & . & 0 & 0 & 0 & . & 0 & 0 \\ a_{12} & a_{22} & 0 & . & 0 & 0 & 0 & . & 0 & 0 \\ 0 & a_{23} & a_{33} & . & 0 & 0 & 0 & . & 0 & 0 \\ . & . & . & . & . & . & . & . & . & . \\ 0 & 0 & 0 & . & a_{ii} & 0 & 0 & . & 0 & 0 \\ 0 & 0 & 0 & . & a_{i,i+1} & a_{i+1.i+1} & 0 & . & 0 & 0 \\ 0 & 0 & 0 & . & 0 & a_{i+1,i+2} & a_{i+2.i+2} & . & 0 & 0 \\ . & . & . & . & . & . & . & . & . & . \\ 0 & 0 & 0 & . & 0 & 0 & 0 & . & a_{299,299} & 0 \\ 0 & 0 & 0 & . & 0 & 0 & 0 & . & a_{299,200} & a_{300,300} \end{vmatrix}$$

where $a_{i,i+1}$ represents the fraction advancing from a given stage class i to age class i+1, and $a_{i,i}$ represents the amount remaining in stage i. The elements $a_{i,i}$ and $a_{i,i+1}$ differ depending on which of the three consumer groups the YOY are in at a given time step. The amount initially in stage i that advances to the next stage, i+1, during a time step is

$$a_{i,i+1} = surv * advance(prey)$$

where *surv* is a fixed constant survival rate for each time step and *advance(prey)* is the fraction of the survivors that advance to the next stage class, which is a function of prey density. The fraction of those initially in stage class i that remain there during the next time step is

$$a_{i,i} = surv * (1 - advance(prey))$$

For simplicity, we assume that the type of prey being consumed does not affect survival, *surv*, but that it does affect advancement, through the functions;

$$advance(zooplankton) = \frac{q_1 Z}{q_2 + Z}$$

$$advance(benthic) = \frac{q_3 M}{q_4 + M}$$

$$advance(preyfish) = \frac{q_5 F}{q_6 + F}$$

where Z, F, and M are the current available biomasses of zooplankton, prey fish, and macroinvertebrates, respectively. The fraction of advance to the next stage depends on the density of available prey. The q_is are constants that can be chosen to corresponding to given assumptions on how the prey biomasses of each type affect advances to the next stage.

In this case it was assumed that YOY perch feeding on fish reach the highest growth rates, followed by those feeding on macroinvertebrates, while planktivorous perch have the lowest growth rates (i.e., $q_3 > q_5 > q_1$). Additionally, the growth rates also depend on the concentrations prey, which are changing through time.

Application of the Markov chain matrix each day changes the numbers of YOY perch in each of the functional consumer groups;

$$B1_{j+1} = surv * (1. - advance(zooplankton)) * B1_{j+1} + surv * advance(zooplankton) * B1_j$$
$$B2_{j+1} = surv * (1. - advance(benthic)) * B2_{j+1} + surv * advance(benthic) * B2_j$$
$$B3_{j+1} = surv * (1. - advance(preyfish)) * B3_{j+1} + surv * advance(preyfish) * B3_j$$

Changing prey densities are described by the dynamic equations:

$$\frac{dZ}{dt} = r_z \left(1 - \frac{Z}{K_z}\right) - \frac{f_z Z \sum_{i=1,300} Weight_i B1_i}{1 + f_z hZ}$$
$$\frac{dM}{dt} = r_m \left(1 - \frac{M}{K_m}\right) - \frac{f_m M \sum_{i=1,300} Weight_i B2_i}{1 + f_m hM}$$
$$\frac{dF}{dt} = r_f \left(1 - \frac{F}{K_f}\right) - \frac{f_f F \sum_{i=1,300} Weight_i B3_i}{1 + f_f hF}$$

where $Weight_i$ is the weight of perch in stage class i, f_z, f_m, and f_f are feeding rates, and $1/h$ is the maximum digestion of biomass per unit time by the perch. r_z, r_m, and r_f are renewal rates of zooplankton, macroinvertebrates and prey-fish, and K_z, K_m, and K_f are the carrying capacities. These equations are transformed to discrete-time difference equations with a time step of a day to be simulated on time steps consistent with the perch.

Finally, perch are assumed to be able to switch prey types when they reach certain threshold stage classes, and switch then with certain probabilities each time step. Perch are assumed to be able to switch prey types when they reach certain threshold stage classes, and switch then with certain probabilities each time step. At this time there are only two switches, from planktivory to macroinvertivory and from macroinvertivory to piscivory.

Stage_transfer_to_inverts = weighth at which planktivores can start to switch to macroinvertivory

Stage_transfer_to_fish = weight at which macroinvertivores can start to switch to piscivory

Fraction_transfer_to_inverts = fraction of planktivores switching to macroinvertivory each time step

Fraction_transfer_to_fish = fraction of macroinvertivores switching to piscivory each time step

Operationally, to transfer perch from one consumer functional group to the next, at each time step, the code iterates through each weight class $Weight_i$, and queries if

$Weight_i > Stage\_transfer\_to\_inverts$

and

$Weight_i > Stage\_transfer\_to\_fish$.

If the first of these is true, and there are planktivorous perch in that weight class, then a number

$B1_{i,move} = Fraction\_transfer\_to\_inverts*B1_i$

are moved to the invertivore subcohort. If the second of these is true, and there are planktivorous perch in that weight class, then a number

$B2_{i,move} = Fraction\_transfer\_to\_inverts*B2_i$

are moved to the piscivore subcohort.

Parameterization

This is a phenomenological model which attempted to describe the perch dynamics as well as that of the prey functional groups. The parameters (listed in Table S4.1) for the three prey functional groups were chosen to approximate the carrying capacities and growth rates of those groups such that, under the conditions of exploitation of perch, they would produce the densities of these groups reported in the Results. To keep fish numbers high, the simulation was performed assuming a ten square meter area rather than the 1 meter square area the results are presented on. For example, macroinvertebrates were assumed to have carrying capacity, K_m of 200 grams dry weight per 10 square meters and a renewal rate, r_m, of 0.3 or 30% per day. In the scenarios, this produced macroinvertebrate densities consistent with those measured. The maximum rate of biomass assimilation per day by the perch was assumed to be 1/h = 0.2, which produced reasonable growth rates of the perch, and the feeding rates, f_z, f_m, and f_f were chosen such that the perch were able to obtain a large fraction of the available prey, though not usually saturated with prey. The coefficients for advance of the fish from one prey type, q_i (i=1,…,6) to the next were chosen to provide reasonable approximation of the progression of the perch through these prey, as we had no a priori estimates of these values. The survival rate of 0.998 was applied across all perch to give reasonable agreement with decline in total mean perch numbers from 14 per square meter to 5 per square meter by the end of the measurements. We acknowledge that these are parameter values chosen phenomenologically to describe the observed phenomena. The model is not intended to be predictive, parameterized on independent data, but to show how the perch dynamics could occur with reasonable values of parameters.

Initial conditions

The YOY perch are assumed to start with a bimodal distribution in which 170 planktivores are distributed among smaller stages.

Table S4.1: Model parameters for the stage-structured model on young-of-the-year perch.

Subject	Symbol	Interpretation	Unit	Parameter value
Survival of perch	*surv*	survival rate	d^{-1}	0.998
Digestion	*h*	inverse maximum digestion	d^{-1} *unit prey biomass*$^{-1}$	5.0
Feeding rate of perch	f_z	on zooplankton	d^{-1}	0.001
	f_m	on macroinvertebrates	d^{-1}	0.0003
	f_f	on prey-fish	d^{-1}	0.0008
Advance of perch from a given stage class *i* to stage class *i*+1	*q1*	constants affecting zooplankivores	*dim'less*	0.2
	q2	advancement to next stage class	*g dW 10m*$^{-2}$	10.0
	q3	constants affecting piscivores	*dim'less*	1.1
	q4	advancement to next stage class	*g dW10m*$^{-2}$	10.0
	q5	constants affecting macroinvertivores	*dim'less*	0.5
	q6	advancement to next stage class	*g dW10m*$^{-2}$	10.0
Zooplankton dynamics	r_z	renewal rate	d^{-1}	0.35
	K_z	carrying capacity	*g dW10m*$^{-2}$	500.0
Macroinvertebrate dynamics	r_m	renewal rate	d^{-1}	0.35
	K_m	carrying capacity	*g dW10m*$^{-2}$	200.0
Prey-fish dynamics	r_f	renewal rate	d^{-1}	0.3
	K_f	carrying capacity	*g dW10m*$^{-2}$	150.0
Transfer of perch at diet shifts	*Stage_transfer_to_inverts*	weight at which planktivores can start to switch to macroinvertivory	*g dW10m*$^{-2}$	1.0
	Fraction_transfer_to_inverts	fraction of planktivores switching to macroinvertivory each time step	d^{-1}	0.04
	Stage_transfer_to_fish	weight at which macroinvertivores can start to switch to piscivory	*g dW10m*$^{-2}$	2.5
	Fraction_transfer_to_fish	fraction of macroinvertivores switching to piscivory each time step	d^{-1}	0.04
