# Supplementary material for: A new mechanistic approach for the further development of a population with established size bimodality
Source: PLoS One. 2017 Jun 26;12(6):e0179339. doi: 10.1371/journal.pone.0179339 (PMC5484486; doi:10.1371/journal.pone.0179339)
Supplement: S1 Table — Given is the weight percentage composition (± standard deviation) of main food resources for perch assigned to different clusters. The calculation of clusters was based on stomach content analysis (Fig 1). Grey shades illustrate to which clusters perch were assigned to for the different dates. For example in June and July two clusters were found, one with perch feeding on prey-fish, the other one with perch consuming a mixed diet of zooplankton and macroinvertebrates. From August onwards perch were assigned to three clusters (consuming fish, macroinvertebrates or zooplankton). MI = macroinvertebrates, ZP = zooplankton, NA = not existent. (PDF) [file pone.0179339.s004.pdf]

S1 Table: Percentage composition of food resources consumed by perch. Given is the weight percentage composition (± standard deviation) of main food resources for perch assigned to different clusters. The calculation of clusters was based on stomach content analysis (Fig 1). Grey shades illustrate to which clusters perch were assigned to for the different dates. For example in June and July two clusters were found, one with perch feeding on prey-fish, the other one with perch consuming a mixed diet of zooplankton and macroinvertebrates. From August onwards perch were assigned to three clusters (consuming fish, macroinvertebrates or zooplankton). MI=macroinvertebrates, ZP=zooplankton, NA= not existent.

	**ZP**		**MI**		**Fish**	
**Cluster 1**						
**22 Jun**	0.02	±0.04	0.00	±0.00	0.98	±0.04
**20 Jul**	0.02	±0.10	0.02	±0.05	0.96	±0.11
**14 Aug**	0.00	±0.01	0.01	±0.03	0.99	±0.03
**13 Sep**	0.07	±0.17	0.01	±0.02	0.92	±0.17
**14 Oct**	0.00	±0.00	0.02	±0.08	0.98	±0.08
**Cluster 2**						
**22 Jun**	0.38	±0.33	0.62	±0.33	0.00	±0.00
**20 Jul**	0.44	±0.34	0.53	±0.31	0.04	±0.11
**14 Aug**	0.10	±0.14	0.90	±0.14	0.00	±0.00
**13 Sep**	0.02	±0.04	0.98	±0.04	0.00	±0.00
**14 Oct**	0.20	±0.20	0.80	±0.20	0.00	±0.00
**Cluster 3**						
**22 Jun**	NA	NA	NA	NA	NA	NA
**20 Jul**	NA	NA	NA	NA	NA	NA
**14 Aug**	0.91	±0.15	0.09	±0.15	0.00	±0.00
**13 Sep**	0.74	±0.24	0.26	±0.24	0.00	±0.00
**14 Oct**	0.99	±0.02	0.01	±0.02	0.00	±0.00
