# Supplementary material for: A new mechanistic approach for the further development of a population with established size bimodality
Source: PLoS One. 2017 Jun 26;12(6):e0179339. doi: 10.1371/journal.pone.0179339 (PMC5484486; doi:10.1371/journal.pone.0179339)
Supplement: S1 Fig — Letters in the fish or directly beside them indicate the food resource consumed. F = fish-eating (piscivorous), MI = macroinvertivorous, ZP = zooplanktivorous. The colours of fish which are explained in the legend box stand for the morphological group fish belong to. At times there might be no overlap of resources consumed and the morphological group. This is because it takes some time for the morph to change after diet shifts. Asterisks indicate an intermediate, but not significantly different morph. E.g., a morph whose traits are in between the one of piscivores and zooplanktivores. (DOCX) [file pone.0179339.s005.docx]

**S1 Fig: Graphical illustration of diet shifts and morphological development of perch from June to October 2006.** Letters in the fish or directly beside them indicate the food resource consumed. F= fish-eating (piscivorous), MI =macroinvertivorous, ZP= zooplanktivorous. The colours of fish which are explained in the legend box stand for the morphological group fish belong to. At times there might be no overlap of resources consumed and the morphological group. This is because it takes some time for the morph to change after diet shifts. Asterisks indicate an intermediate, but not significantly different morph. E.g., a morph whose traits are in between the one of piscivores and zooplanktivores.
